# Generation of micro- and nano-morphologies on a stainless steel surface irradiated with 257 nm femtosecond laser pulses

**DOI:** 10.1039/c8ra01774c

**Published:** 2018-04-30

**Authors:** Fotis Fraggelakis, Girolamo Mincuzzi, Inka Manek-Hönninger, John Lopez, Rainer Kling

**Affiliations:** ALPhANOV, Technological Centre for Optics and Lasers, Optic Institute of Aquitaine Rue F. Mitterrand, 33400 Talence France fotis.fraggelakis@alphanov.com; CELIA University of Bordeaux-CNRS-CEA UMR5107 33405 Talence France

## Abstract

Surface structuring by femtosecond lasers has emerged as an efficient tool to functionalize the surfaces of various solid materials. Laser induced periodic surface structures (LIPSS) can drastically impact the wetting, friction and optical properties of the surface depending on the size, aspect ratio and period of the structures. Morphological characteristics in the nanoscale, such as nano roughness, contributing to a hierarchical surface formation are considered to have a significant impact on those properties. In this study, we demonstrate for the first time to our knowledge the feasibility of inducing ripples and spikes utilizing a 257 nm femtosecond laser. LIPSS with a period smaller than 200 nm were realised. Furthermore, we show the evolution of those structures into conical spikes for this wavelength, and we provide an interpretation on their formation. Finally, we show that sub 200 nm LIPSS can create subwavelength gratings providing non-angular dependent light reflection and non-periodic morphologies showing super hydrophobic behaviour.

## Abstract 2

Ripples (150 nm period) and spikes (few microns size) were shown for the first time utilizing a 257 nm femtosecond laser.

## Graphical abstract

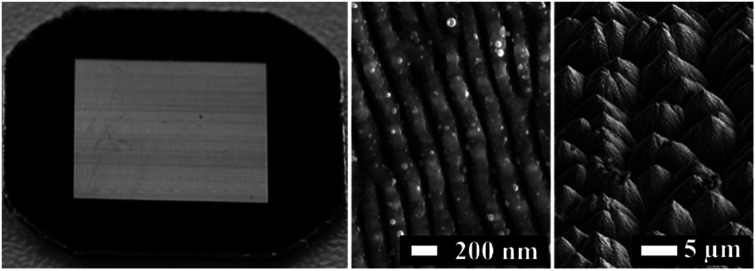

## Introduction

Laser surface structuring has proven to be a powerful technology to modify the surface properties of different kinds of solids without the use of coatings. Femtosecond lasers made it possible to structure the surface of various materials such as metals,^1^ ceramics,^2,3^ dielectrics^4,5^ and semiconductors.^6^ These modifications are attributed to be linked to both laser induced structures and chemical surface composition changes during the irradiation. Laser induced structures, such as periodical formations like grooves and spikes, are reported to induce remarkable modifications of surface wetting,^7–10^ tribological,^11^ and optical properties.^12–14^ Recent studies showed that LIPSS can also have an influence on biofilm adhesion on steel surfaces^15^ and the growth of body cells.^16^ Nowadays, such laser induced structures can be applied to create specific desired surface properties of industrial or domestic devices as well as for medical applications such as implants and surgery equipment and are therefore of very high interest.

LIPSS morphology has been attributed to key process parameters such as the wavelength and the incident angle of the laser beam^17^ as well as the fluence and the number of pulses.^18^ Even though a complete theory to describe LIPSS formation has not been established yet, some of the proposed mechanisms to explain the formation of these morphologies are surface plasmon coupling with the incident light,^17^ surface self-organization^19^ and the coupling of the incident electromagnetic wave with the surface roughness.^20^ Concerning the formation mechanism of spikes, *i.e.* conical quasi periodical structures that can be induced on metals^1,21^ and semiconductors,^22^ several studies have been carried out on controlling their size^21,23^ and two distinct mechanisms are proposed to lead to spike formation. For metals irradiated with femtosecond pulses with fluence values close to the ablation threshold, the interpretation of spike formation is based on the ablation around surface defects. The light which is deflected from the top of defects ablates the surface around the defects and thus leads to conical shaping after an accumulation of a few hundreds of pulses on the surface.^24^ For higher fluence values, the spike formation could be ascribed to thermofluidic movements resulting from thermal gradients^23^ in a similar way as described for silicon.^22^ Indeed, for silicon irradiated with infrared or near-infrared femtosecond pulses, many contributions on the study of laser induced structures can be found in literature.^25–27^ However, a few studies have been carried out on micro- and nano-formation on metallic surfaces^28,29^ with femtosecond laser and in particular on stainless steel.^30–32^

In this work, we report for the first time to our knowledge on laser induced formation of sub 200 nm morphologies on a stainless steel surface using femtosecond laser pulses in the deep UV at 257 nm. LIPSS formation under UV femtosecond irradiation was studied in diamond^33^ and silicon.^34,35^ We provide novel and valuable data about LSFL (low spatial frequency LIPSS^36^) with sub 200 nm period and HSFL (high spatial frequency LIPSS^37,38^) with sub 100 nm period, much lower than those reported up to date for this material.^39^ Moreover, we show a sequence of the evolution of those surface structures, from HSFL into spikes under successive pulse irradiation, which is providing an interpretation of the spike formation mechanism. We believe that these results will contribute to a better understanding of LIPSS formation on stainless steel as well as of the mechanism of groove and spike creation. Finally, we demonstrate possible applications of two morphologies, a surface with LSFL, which can act as a subwavelength grating, and a surface structure covered with laser induced roughness exhibiting superhydrophobic properties.

## Experimental

A commercial ultrafast fiber laser system (Satsuma HP^3^, Amplitude Systèmes) emitting in the near infrared (*λ* = 1030 nm) with a maximum output power of 40 W and delivering ultra-short pulses of 350 fs was utilized as laser source. The delivered laser beam was linearly polarized with a Gaussian intensity profile. An external module was installed to produce the deep UV radiation at 257 nm by fourth harmonic generation. We expanded the beam by a factor of 2 and focalized it with a 160 mm f-theta lens. The spot diameter on the sample was estimated to 18 μm (2*ω*_0_) as described by J. M. Liu.^40^ With this setup, we structured the surface of 316 L stainless steel of 0.5 mm thickness by scanning over the sample surface using a Galvo scanner (RAYLASE Turboscan). To evaluate a broad range of parameters we processed a matrix of 1 mm × 3 mm rectangles on each sample.

In this study, we varied the parameters of the fluence (*Φ*), the number of scans (*N*) and the pulses per spot (pps). The latter is defined as the average number of pulses irradiated in a dimensionless spot on the surface within a single scan and corresponds to the overlap (pps). The pps_tot_, defined as pps*N*, is a variable used to describe the total amount of pulses irradiated on the surface.

Throughout the experimentsthe repetition rate has been kept constant at 250 kHz. The fluence was varied between *Φ*_Low_ = 0.11 J cm^−2^ and *Φ*_High_ = 0.42 J cm^−2^ by increasing the laser pulse energy from 0.28 μJ to 1.08 μJ. The overlap was chosen between 2 pps and 100 pps, and the number of successive scans *N* was varied between *N* = 1 and *N* = 50. Finally, the distance between two successive scanning lines (hatch) was fixed to *d* = 5 μm.

After processing, the material was cleaned in an ultrasonic acetone bath before SEM characterization in order to remove the ablation dust produced during the laser irradiation. The surface morphology analyses were carried out utilizing a CSEM-FEG INSPECT 50 scanning electronic microscope. For these morphology analyses and the Fourier transformation images, an open source software (Gwyddion) was employed.

## Results and discussion

### Surface evolution. Ripple, groove and spike formation

Femtosecond laser irradiation can enable the formation of periodic structures such as ripples, grooves and spikes. For a given material the morphology of the incident structures *e.g.* the period for the LSFL, HSFL and grooves, as well as the diameter and the aspect ratio of spikes depend on the laser parameters, especially on the wavelength, the fluence and the pps_tot_.

**Fig. 1:**
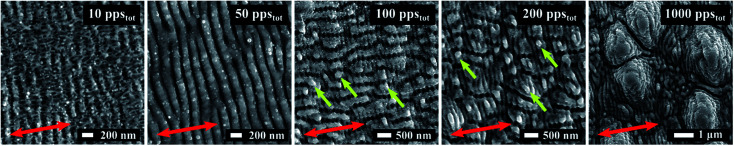
SEM images of stainless steel surface showing different morphologies obtained by UV LIPSS. The red arrows indicate the polarization orientation. The total number of pulses is indicated by the pps_tot_ value in each image. Fluence was fixed to *Φ*_Low_ = 0.11 J cm^−2^. HFSL are formed for pps_tot_ = 10, ripples (LFSL) for 50 pps_tot_, grooves for pps_tot_ = 100, pre-spikes for pps_tot_ = 200, and inhomogeneous spikes for 1000 pps_tot_. The yellow arrows point to the small protrusions formed in the edge of the groove for 100 pps_tot_ and in the center of the rim for 200 pps_tot_.

**Fig. 2:**
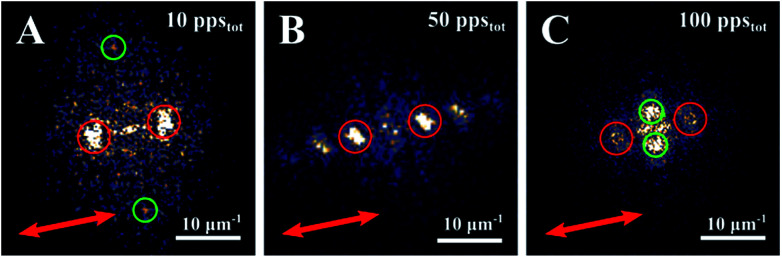
FT diagrams corresponding to the SEM images of Fig. 1 as indicated by the pps_tot_ value. The red arrows indicate the polarization orientation. Ripple periodicities are marked with red dotted circles in (A)–(C). Green circles indicate HFLS in image (A) and groove structure in image (C).

In this paragraph, we report on the obtained morphological characteristics on stainless steel employing our above described experimental setup under variation of pps_tot_ while the fluence was fixed at *Φ*_Low_ = 0.11 J cm^−2^. The resulting morphologies are shown in Fig. 1. Here, a progressive evolution of the surface structures is presented for increasing pps_tot_, from HSFL (pps_tot_ = 10) to an inhomogeneous spike formation (pps_tot_ = 1000). The various periods of the structures were determined using Fourier transformation (FT). The FT images corresponding to the SEM images in Fig. 1 for pps_tot_ = 10, pps_tot_ = 50 and pps_tot_ = 100, are presented in Fig. 2A, B and C, respectively.

In detail for pps_tot_ = 10, HSFL were obtained oriented parallel to the polarization direction (indicated with a red arrow) with a period as short as *Λ*_HSFL_ = 76 ± 2 nm (Fig. 2A) while at this point, ripples start to form on the surface. For pps_tot_ = 50 the ripples, oriented perpendicular to the laser polarization (leading to LSFR), become well defined with *Λ*_LSFL_ = 153 ± 4 nm (Fig. 2B). For pps_tot_ = 100 the predominant morphology on the surface consists of shallow structures oriented parallel to the laser polarization but with a period almost 6 times longer than for HSFL. Those structures are often mentioned as grooves and they are considered as an intermediate structure between ripples and spikes.^22^ Similar formations on the same material were also reported using different wavelengths.^23^ For the given conditions a period of *Λ*_groove_ = 426 ± 7 nm (Fig. 2C) could be extracted. In some cases, small protrusions are formed at the edges of the grooves, indicated with yellow arrows in Fig. 1 for pps_tot_ = 50. Under further irradiation (Fig. 1 pps_tot_ = 200) the number of protrusions, marked with yellow arrows, is higher and the grooves are shorter in length.

Elliptical, submicron, spiky protrusions with low aspect ratio are formed around those protrusions. We assume that their formation mechanism is similar to the one described by Tsibidis *et al.* in ref. 22 for silicon. In their work, simulations show that the spike formation process can be explained as a result of Marangoni flow over lateral and in depth temperature gradients. This model could possibly be applied to our results on stainless steel.

However, the penetration depth which determines the width of the absorbing zone is expected to be shorter for UV than in IR.^41^ Thus the residual thermal gradient which is considered to be the dominant mechanism for groove and spike formation should differ substantially from the one described in ref. 22. Consequently, the groove depth, the ratio between ripples and grooves as well as the aspect ratio between spike height and width is expected to be smaller than in silicon.

**Fig. 3:**
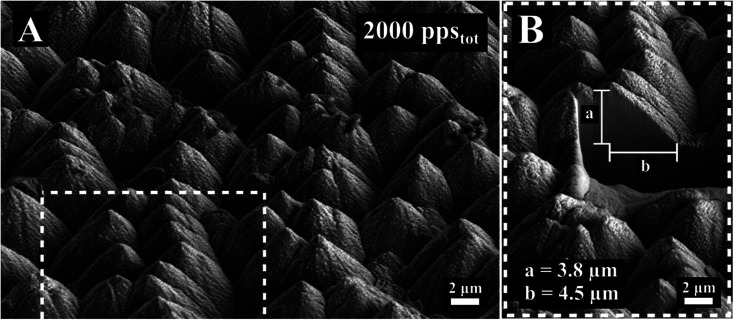
(A) SEM image of the surface morphology obtained for 2000 pps_tot_. (B) detail of (A) showing the cross section of spikes.

For increasing pps_tot_ the surface changes radically. The inhomogeneity increases and conical formations start to develop on the surface (Fig. 1, 1000 pps_tot_). These conical formations densify and grow as the number of pulses increases. As shown in Fig. 3, spikes become the predominant shape on the surface for 2000 pps_tot_. In Fig. 3B a cross section of conical spike formation gives additional information about the size and the aspect ratio of the features. In this particular case, the height was estimated to be 3.8 μm and the half of the base 4.5 μm, relatively smaller compared to IR spike formation on steel.^23,42^ Lastly, the mechanism of their growth should be attributed to ablation rather than hydrodynamical movement of molten material. Spikes appear randomly on the surface (Fig. 1, 1000 pps_tot_) and as the pps_tot_ increases they are densified in the surface (Fig. 3). A similar evolution of stainless steel surface has been observed for IR femtosecond pulse irradiation and is attributed to anisotropy of beam absorption due to surface defects.^24^

### The impact of fluence

**Fig. 4:**
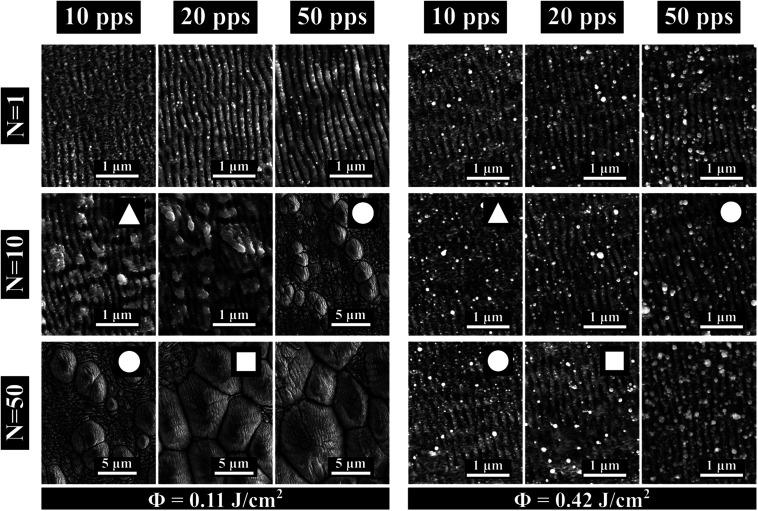
SEM images of stainless steel surface obtained with two fluence values, *Φ*_Low_ = 0.11 J cm^−2^ and *Φ*_High_ = 0.42 J cm^−2^. Different overlaps (pps) and number of scans (*N*) were studied. Morphologies obtained after irradiation with the same dose (same number of pulses pps_tot_ with the same *Φ*) are indicated with the same symbol ▲: pps_tot_ = 100, ●: pps_tot_ = 500, ■: pps_tot_ = 1000.

Here we study the effects for two different fluences, *Φ*_Low_ = 0.11 J cm^−2^ and *Φ*_High_ = 0.42 J cm^−2^ on surface morphology. For each fluence value we vary the overlap between pps = 10 and pps = 100 and the number of scans between *N* = 1 and *N* = 50. The results are illustrated in Fig. 4. Same pps_tot_ values are indicated with different symbols ▲: pps_tot_ = 100, ●: pps_tot_ = 500, ■: pps_tot_ = 1000.

For *Φ*_Low_ as the pps_tot_ increases, independently of the irradiation strategy, the structures evolve from ripples into grooves and conical spikes. Surface structuration obtained with the same pps_tot_ exhibits only minor differences for different irradiation strategies.

Interestingly, when the fluence is set to *Φ*_High_ the obtained surface morphologies differ substantially from the ones obtained for *Φ*_Low_. Here (Fig. 4*Φ*_High_) we no longer observe a diversity of the surface structures.

Indeed, in this case the surface doesn't change as the pps_tot_ increases and is dominated in all cases by inhomogeneous ripples. This behaviour leads us to the conclusion that for this fluence value a significant part of the surface which participates in the interaction is ablated. Enhanced ablation for increasing fluence has been observed in metals irradiated with IR femtosecond pulses^43^ but for much higher fluences, in the range of 2–10 J cm^−2^. Furthermore, multi-pulse LIPSS formation is the cumulative result of the coupling of the incident pulse with the pre-existing surface roughness.^22^ The pps value defines the spatial relation between the crater which is formed by the previous pulse and the incident pulse intensity distribution. Therefore, variations of the pps value can affect the resulting morphology even when the pps_tot_ value is the same. As we can see in Fig. 4 this phenomenon is negligible for *Φ*_Low_ but intensified for *Φ*_High_. In that case we expect that the crater formed by each pulse is bigger and thus has greater impact on the irradiation.

### Macroscopic visual appearance and wettability properties

**Fig. 5:**
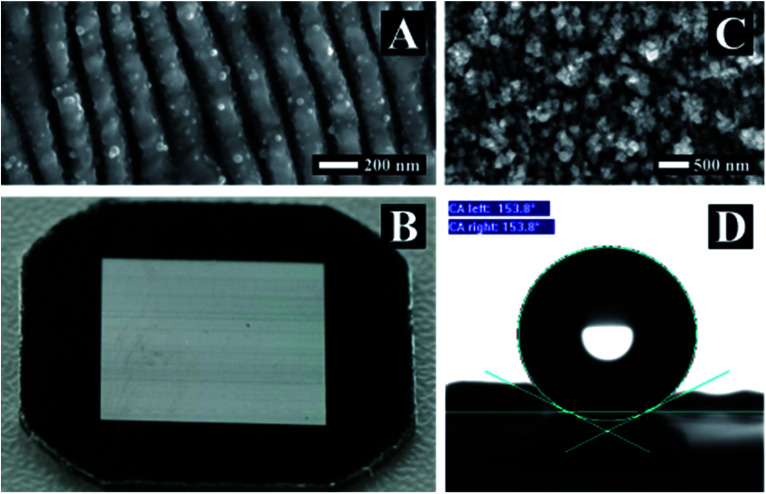
Applications on stainless steel surface. (A) SEM image of rippled surface corresponding to (B). (B) Visual appearance of a stainless steel surface 1 cm^2^ textured with ripples. Picture was taken under angle. (C) SEM image of surface processed with *Φ*_High_ = 0.42 J cm^−2^, *N* = 1 and pps = 1000. (D) Contact angle measurement of a superhydrophobic 5 mm × 5 mm steel surface with nano-roughness texture corresponding to (C).

The macroscopic visual appearance and wettability properties of the surface were also considered. Here we present two of the obtained morphologies that are featuring interesting properties. A surface textured homogeneously with LIPSS over an area of a cm^2^ and a surface with high nano-roughness are shown in Fig. 5B and C, respectively.

Periodic structures with one or two symmetry axes and periods smaller than the incident wavelength behave as subwavelength gratings.^44^ Such subwavelength gratings have a variety of applications and can be utilized as polarization converters,^45^ broadband mirrors,^46^ and in the case of two dimensional surface structures the surface obtains antireflection properties.^47,48^ Nevertheless, the structures should have a period close to or smaller than a couple of hundreds of nanometres and so far it wasn't possible to produce these structures with lasers. We were able to texture homogeneously an area of a cm^2^ with a ripple period in the order of *Λ*_LSFL_ = 150 nm (Fig. 5A). The surface's optical behaviour is illustrated in Fig. 5B. All visible wavelengths are reflected at the same angle making the surface appearing white while the non-processed area appears black.

Surface hydrophobicity or super hydrophobicity is another desired surface property that can be laser induced either by direct laser writing^49^ or as a result of micro spike texturing.^50^ It can be argued that wetting properties are an outcome of the combined effect of microstructure and nano-roughness as well as from changes in chemical composition of the surface.^9^ Interestingly, the nano-roughness structuration (Fig. 5C) exhibits by itself superhydrophobic behaviour and a contact angle of 154° was measured. The structure was produced by irradiating the surface with *Φ*_High_, *N* = 1 and pps = 1000 and was cleaned in an acetone ultrasonic bath for several minutes to remove dust.

## Conclusions

We report for the first time to our knowledge the generation of ripples, grooves and spikes with ultraviolet femtosecond pulses at 257 nm. We provide a detailed description and interpretation of the transition between the different structures and we describe the connection between the structure and the laser polarization. Furthermore, we report on the fabrication of a subwavelength grating by a homogeneous surface structuration of ripples on stainless steel over a cm^2^. Finally, we produced a superhydrophobic surface which results only of laser induced nano roughness. We believe that our results can contribute to a better understanding of the LIPSS formation process and extend the range of applications in the functionalization of surfaces.

## Conflicts of interest

There are no conflicts to declare.

## Supplementary Material
